# Targeted delivery of nerve growth factor to the cholinergic basal forebrain of Alzheimer’s disease patients: application of a second-generation encapsulated cell biodelivery device

**DOI:** 10.1186/s13195-016-0195-9

**Published:** 2016-07-07

**Authors:** Helga Eyjolfsdottir, Maria Eriksdotter, Bengt Linderoth, Göran Lind, Bengt Juliusson, Philip Kusk, Ove Almkvist, Niels Andreasen, Kaj Blennow, Daniel Ferreira, Eric Westman, Inger Nennesmo, Azadeh Karami, Taher Darreh-Shori, Ahmadul Kadir, Agneta Nordberg, Erik Sundström, Lars-Olof Wahlund, Anders Wall, Maria Wiberg, Bengt Winblad, Åke Seiger, Lars Wahlberg, Per Almqvist

**Affiliations:** Department of Neurobiology, Care Sciences and Society, Karolinska Institutet, 171 77 Stockholm, Sweden; Department of Geriatrics, Karolinska University Hospital, Huddinge, 171 76 Stockholm, Sweden; Department of Clinical Neuroscience, Karolinska Institutet, 171 77 Stockholm, Sweden; Department of Neurosurgery, Karolinska University Hospital Solna, Building R3:02, 171 76 Stockholm, Sweden; NsGene Inc., 225 Chapman Street, Providence, RI 02905-4533 USA; Clinical Neurochemistry Laboratory, Department of Clinical Neuroscience, University of Gothenburg, 41345 Gothenburg, Sweden; Department of Laboratory Medicine, Section of Pathology, Karolinska University Hospital, 171 76 Stockholm, Sweden; Stiftelsen Stockholms Sjukhem, Mariebergsgatan 22, 112 35 Stockholm, Sweden; Department of Surgical Sciences, Section of Nuclear Medicine and PET, Uppsala University Hospital, 75185 Uppsala, Sweden; Department of Clinical Science, Intervention and Technology, Division of Medical Imaging and Technology, Karolinska Institutet, 171 77 Stockholm, Sweden; Department of Radiology, Karolinska University Hospital, 171 76 Stockholm, Sweden

**Keywords:** Nerve growth factor, Alzheimer’s disease, Regenerative medicine, Encapsulated cell biodelivery

## Abstract

**Background:**

Targeted delivery of nerve growth factor (NGF) has emerged as a potential therapy for Alzheimer’s disease (AD) due to its regenerative effects on basal forebrain cholinergic neurons. This hypothesis has been tested in patients with AD using encapsulated cell biodelivery of NGF (NGF-ECB) in a first-in-human study. We report our results from a third-dose cohort of patients receiving second-generation NGF-ECB implants with improved NGF secretion.

**Methods:**

Four patients with mild to moderate AD were recruited to participate in an open-label, phase Ib dose escalation study with a 6-month duration. Each patient underwent stereotactic implant surgery with four NGF-ECB implants targeted at the cholinergic basal forebrain. The NGF secretion of the second-generation implants was improved by using the Sleeping Beauty transposon gene expression technology and an improved three-dimensional internal scaffolding, resulting in production of about 10 ng NGF/device/day.

**Results:**

All patients underwent successful implant procedures without complications, and all patients completed the study, including implant removal after 6 months. Upon removal, 13 of 16 implants released NGF, 8 implants released NGF at the same rate or higher than before the implant procedure, and 3 implants failed to release detectable amounts of NGF. Of 16 adverse events, none was NGF-, or implant-related. Changes from baseline values of cholinergic markers in cerebrospinal fluid (CSF) correlated with cortical nicotinic receptor expression and Mini Mental State Examination score. Levels of neurofilament light chain (NFL) protein increased in CSF after NGF-ECB implant, while glial fibrillary acidic protein (GFAP) remained stable.

**Conclusions:**

The data derived from this patient cohort demonstrate the safety and tolerability of sustained NGF release by a second-generation NGF-ECB implant to the basal forebrain, with uneventful surgical implant and removal of NGF-ECB implants in a new dosing cohort of four patients with AD.

**Trial registration:**

ClinicalTrials.gov identifier: NCT01163825. Registered on 14 Jul 2010.

## Background

An estimated 44 million individuals worldwide have dementia, the majority of whom have Alzheimer’s disease (AD) [1]. Common to patients with AD is an early loss of basal forebrain cholinergic neurons, resulting in degeneration of cortical and hippocampal projections [2]. Pre-clinical and clinical observations indicate a strong association between cholinergic dysfunction and cognitive impairment in patients with AD [3, 4] that lead to the development of cholinesterase inhibitors (ChEIs).

Cholinergic degeneration is, in part, the result of impaired nerve growth factor (NGF) signalling and transport to the basal forebrain cholinergic neurons [5]. NGF is neuroprotective for basal forebrain cholinergic neurons and reduces cognitive deficits in animal models of AD [6, 7]. However, NGF does not cross the blood-brain barrier, owing to its chemical properties, and requires targeted delivery to the brain to have a biological effect. A constraint has been the difficulty in identifying a mode of administration that selectively provides long-term delivery of NGF in sufficient quantities to target areas of the brain to achieve clinical efficacy. Several strategies of direct delivery have been investigated, including direct central nervous system (CNS) infusion [8], intra-nasal administration [9], gene therapy approaches [10] and cell-based delivery using stem cells [11]. Each approach has advantages as well as practical and technical restrictions limiting further development. Intra-ventricular infusion of NGF in three patients with AD resulted in positive effects on nicotinic binding and cerebral blood [12]. However, the patients developed significant side effects, such as back pain and weight loss, making this route of NGF administration unsuitable [8]. Animal studies later indicated that these adverse events were caused by NGF binding to dorsal root ganglion neurons and the hypothalamus, eliciting a pain response and weight loss [13]. None of these adverse effects was seen following intra-parenchymal NGF administration in rodents [14]. The safety of intra-parenchymal NGF delivery was further substantiated by the fact that no pain was experienced by patients with Parkinson’s disease who were receiving NGF infusion directly into the striatum [15] or by cognitively impaired primates following transplant of NGF-secreting fibroblasts to the basal forebrain [16].

This latter approach was tested in a clinical trial of ex vivo gene delivery of NGF to the basal forebrain in patients with AD, and the treatment was well-tolerated, without any adverse effects being attributed to NGF [10]. NGF delivery via CERE-110, an adeno-associated virus-based gene delivery vector encoding for human NGF, showed sustained NGF expression in rodents for 12 months [17], and it was found to be safe and well-tolerated in patients with AD in a 2-year pilot study [18]. However, with this in vivo gene therapy approach, there are certain limitations and safety concerns, including permanent genetic modification of brain cells and the inability to control or stop release of bioactive substance.

To circumvent these limitations, we applied an encapsulated cell technology (encapsulated cell biodelivery [ECB]) [19] developed by NsGene A/S, Ballerup, Denmark. The implant is a catheter-like device containing a genetically engineered, NGF-secreting, human cell line housed behind a semi-permeable, hollow fibre membrane at its tip. This encapsulation is immunoisolatory and protects the transplanted allogeneic cell line from host immune system rejection while nutrients, oxygen and the NGF produced can diffuse freely across the wall of the capsule. An advantage of the ECB technology over traditional ex vivo or in vivo gene therapy approaches is that the NGF delivery can be stopped by removing the device that contains the genetically modified NGF-producing cells from the brain.

We explored ECB of NGF (NGF-ECB) in six patients with AD in a phase Ib study in three dose cohorts, and results from the third cohort are reported here. Results from earlier dose cohorts were previously reported [20, 21], and they demonstrated safety and feasibility, lower rates of brain atrophy, stable cognitive function, and improvement in cerebrospinal fluid (CSF) cholinergic markers in a subset of patients [22, 23]. NGF release upon explant was low, albeit detectable, and viable cells were observed. We developed a second-generation NGF-ECB implant to achieve increased sustained release of NGF that was validated in pre-clinical animal studies [24]. The primary objective of the present study was to assess the safety and tolerability of device and cell-line combination for a second-generation NGF-ECB implant. Secondary objectives included analyses of cognition and biomarkers.

## Methods

### Study design

**Fig. 1:**
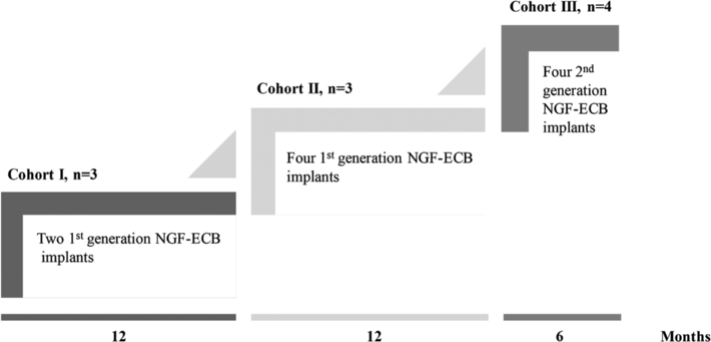
Schematic representation of the dose escalation regimen of the study

We report the results of an open-label dose escalation phase Ib study of targeted delivery of NGF to the cholinergic basal forebrain in four patients with mild to moderate AD for a 6-month duration. The first two dosing cohorts of three patients each received implants of a first-generation NGF-ECB device (NsG0202) (Fig. 1) [20]. The present dose cohort consisted of four patients who received implants of a second-generation NGF-ECB device left in place for 6 months. The aims of the study were to assess safety and tolerability, device performance, clinical efficacy and AD biomarkers.

### Participants

Patients were recruited and enrolled into the study from the Memory Clinic, Karolinska University Hospital, Huddinge, Sweden. The primary inclusion criteria were (1) a probable diagnosis of mild to moderate AD according to the National Institute of Neurological and Communicative Disorders and Stroke-Alzheimer’s Disease and Related Disorders Association criteria [25], (2) aged 50–80 years, (3) Mini Mental State Examination (MMSE) [26] score of 15–24, (4) living at home with a caregiver and (5) stable treatment with ChEIs for at least 3 months before enrolment as well as during the study. Exclusion criteria included (1) ongoing medical and/or psychiatric conditions, (2) antipsychotic drug treatment and (3) smoking (so as not to interfere with nicotinic binding on positron emission tomography [PET]; see paragraph Positron Emission Tomography). To exclude significant neurological conditions contraindicating surgery, we performed brain magnetic resonance imaging (MRI) for all patients. A clinician (HE) established the diagnosis of probable AD based on the above-described criteria, which was reviewed by the principal investigator (ME). Patients and caregivers gave us written informed consent prior to enrolment. The Swedish Medical Products Agency and the Regional Human Ethics Committee of Stockholm approved the study.

### NGF-ECB implant

The NsG0202.1 implant is a gene therapy medicinal product consisting of NGC-0211 cells expressing human NGF, and it is encapsulated in a pre-assembled device. The cells are derived from the ARPE-19 human retinal pigment epithelial (RPE) cell line (American Type Culture Collection, Manassas, VA, USA), transfected, and modified with the human *NGF* gene as described previously [24]. Briefly, ARPE-19 cells were co-transfected with separate plasmids coding for human NGF and a Sleeping Beauty (SB) transposase [27]. The NGF plasmid contains the neomycin antibiotic resistance gene, and several stable clones were selected and tested in vitro and in vivo. The NGC-0211 cell line showed stable long-term performance in experimental devices tested in animal models and was selected as the clinical cell line. The cell line was tested for safety according to regulatory guidelines, and it was characterized with respect to secreted NGF bioactivity, processing and amino acid sequence before clinical application.

**Fig. 2:**
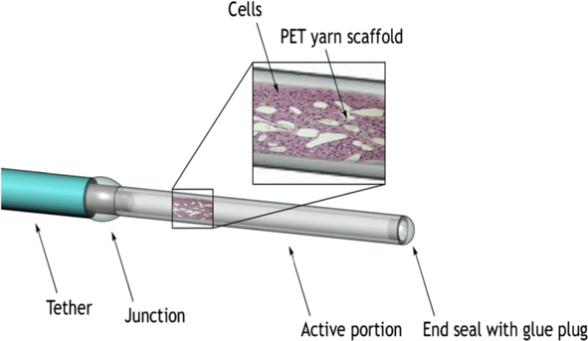
Schematic representation of the NsG0202.1 device

The NsG0202.1 implant was produced under Good Manufacturing Practice (GMP), as previously described for NsG0202 [21]. Briefly, a 150-mm-long, 1-mm-wide, hollow, barium-impregnated polyurethane tether (Carbothane; Lubrizol Corp., Wickliffe, OH, USA) was attached to an 11-mm-long, polyethersulphone, hollow fibre membrane (AkzoNobel/Membrana/3 M, Wuppertal, Germany) via a titanium linker (Heraeus Materials, S.A., Yverdon-les-Bains, Switzerland). The semi-permeable hollow fibre membrane has an outer diameter of 0.72 mm and a mean molecular weight cut-off of 280 kDa. The pre-assembled and gamma-sterilized device was in turn filled with GMP banked NGC-0211 cells a few weeks before implant. A shelf-life of 4.5 weeks after product release was validated to allow ample time for the implant procedure. The polyvinyl alcohol sponge matrix used in the first-generation NsG0202 device was replaced with a polyester terephthalate yarn matrix as an internal cell-supportive scaffold (Fig. 2). This matrix allowed for improved cell adherence, cell survival, and manufacturability. NsG0202.1 implants were tested for safety and toxicology in pre-clinical animal studies [24]. They were kept in sealed, sterile containers filled with human endothelial serum-free medium (Life Technologies, Carlsbad, CA, USA) at 37 °C for up to 4.5 weeks and tested for sterility, mycoplasma, cell leakage and NGF production. On the basis of in vitro analysis, implants releasing NGF within a range of 7.4–10.8 ng NGF/device/24 h were selected for implant.

### Surgical procedures

The details of the surgical procedure and the description of the technical platform with the first-generation NGF-ECB device were reported in 2012 [21]. Briefly, patients underwent MRI-guided stereotactic implant procedures with two NsG0202.1 implants in each hemisphere while under general anaesthesia. The anatomical targets were the centre of the nucleus basalis of Meynert (Ch4) and the vertical limb of the diagonal band of Broca (Ch2) in the basal forebrain. Definition of the anatomical targets was performed using brain atlas coordinates [28] and modified according to each patient’s brain anatomy. The patients underwent cranial computed tomography (CT) immediately after the surgical procedure for safety assessment and documentation of implant positions by using an image fusion technique (Elekta AB, Stockholm, Sweden). At the end of the study, all four patients underwent surgical removal of the implants under general anaesthesia according to the protocol described previously [20].

### Implant function

During the explant procedure, the tethers of retrieved implants were cut and the cell-containing capsules were placed in 1 ml of human endothelial serum-free medium (Life Technologies) and analysed for NGF as previously described [21]. Results were expressed in nanograms NGF per 24 h after extrapolation for incubation time. For evaluation of cell survival and morphology, cell capsules were fixed in a 4 % formalin solution, dehydrated in a graded ethanol series, and embedded in historesin (Leica Microsystems, Wetzlar, Germany). Sections (5 μm) were mounted on slides coated with poly-l-lysine and stained with hematoxylin and eosin (VWR Bie & Berntsen A/S, Søborg, Denmark).

### Histopathology

A cortical brain biopsy was performed at the entry point of one of four implants (at a cortical gyrus of the frontal lobe, 20 mm anterior to the coronary suture and 30–50 mm lateral to midline) in each patient. Tissue was immediately fixed in 4 % paraformaldehyde solution, and 5-μm-thick paraffin sections were cut and stained with antibodies against hyperphosphorylated tau (clone AT-8, 1:500 dilution; Fujirebio Europe, Zwijnaarde, Belgium) and β-amyloid (clone 6F/3D, 1:400 dilution; DakoCytomation, Glostrup, Denmark). The product was visualized using the Zymed Lab-SA detection system (Life Technologies).

### Clinical assessments

The primary outcome measures were safety and tolerability of NsG0202.1 as assessed by reports of adverse events (AEs), vital signs, concomitant medications, pain (assessed with a visual analogue scale), body mass index and laboratory tests. Physical and neurological examinations and cranial MRI were performed at baseline and at 3 and 6 months to verify implant positions and to screen for signs of haemorrhage, infarction, infection, oedema or necrosis. Secondary outcome measures included cognition (assessed with MMSE and Alzheimer’s Disease Assessment Scale-Cognitive subscale [ADAS-Cog] [29]), CSF biomarkers, brain atrophy seen on MRI scans, and biomarkers of cholinergic function (nicotinic receptor binding on PET and CSF cholinergic biomarkers) at baseline and at 3 and 6 months.

### Magnetic resonance imaging

All patients were scanned on a 1.5-T Magnetom Avanto MRI scanner (Siemens, Erlangen, Germany) as previously described [22]. Full brain and skull coverage was obtained, and quality control was carried out on all images according to previously published criteria [30]. Data processing was performed with FreeSurfer 5.1.0 software (http://surfer.nmr.mgh.harvard.edu/) as detailed elsewhere [22]. Visual quality control was performed, and an index of global brain atrophy was calculated as follows: brain volume (BV)/CSF index = (total grey matter volume + total white matter volume)/total CSF volume. Lower values of the BV/CSF index represent greater brain atrophy.

### Positron emission tomography

Measurements of regional cerebral blood flow and cerebral nicotinic receptor binding were performed using a dual-tracer protocol including ^15^O-water and (*S*)-*N*-[^11^C-methyl]nicotine [31]. A flow-compensated parameter, K*_2_, was calculated from the rate constant K_2_ for nicotine divided by cerebral blood flow [32]. A low K*_2_ value indicates high ^11^C-nicotinic binding. ^11^C-nicotinic binding was expressed as a percentage increase from baseline value, calculated by using the following formula: 100 − % K*_2_ = 100 − (K_2_(f)*/K_2_(b)*100), where f and b indicate the time of observation (3 and 6 months) and baseline, respectively. No baseline data were present for patient 1, owing to technical difficulties with the PET nicotine tracer. For the purpose of calculating change from baseline for this patient, the mean value for baseline ^11^C-nicotinic binding for the other three patients was calculated and extrapolated as the baseline value for patient 1.

### Laboratory tests

Routine blood (serum) and CSF samples were collected at baseline and at 3 and 6 months. Serum and CSF samples were aliquoted into polypropylene tubes and stored at −80 °C until analysis. Analyses of CSF amyloid-beta peptide 1–42 (Aβ_1–42_, Aβ), total tau (t-tau) and phosphorylated tau 181 (p-tau) were performed with xMAP technology using the INNO-BIA AlzBio3 kit (Fujirebio Europe) as previously described [33]. CSF levels of neurofilament light chain protein (NFL) and glial fibrillary acidic protein (GFAP) were analysed using previously described enzyme-linked immunosorbent assay (ELISA) methods [34, 35]. CSF activity of acetylcholinesterase (AChE) was assessed by using the modified Ellman’s colorimetric assay, and protein levels of AChE were assessed by employing a functional ELISA as described previously [36]. Activity and protein levels in CSF of choline acetyltransferase (ChAT) were measured by using a recently developed colorimetric assay [37]. All NGF-ECB implants were analysed for NGF release and cell viability by ELISA and histology. An assay for NGF (ELISA kit, minimal detection level 31 pg/ml; R&D Systems, Minneapolis, MN, USA) and ELISA for detection of anti-NGF antibodies in serum and CSF were validated by Vecura AB, Stockholm, Sweden, according to Good Laboratory Practice.

### Statistical analyses

Statistical analyses were performed at an individual level using each patient’s own baseline value as a control. The Wilcoxon signed-rank test was used to compare baseline data with 3- and 6-month post-operative time points. Spearman’s rank correlations were used to investigate relationships between variables. A *p* value <0.05 was deemed significant in all statistical tests. The IBM SPSS 22.0 (IBM, Armonk, NY, USA) and Statistica 7.0 (StatSoft, Tulsa, OK, USA) software packages were used for the analyses.

## Results

### Patient population

The study population included four patients (three men and one woman). The median age of the group was 64 years (range 57–68), and the median MMSE and ADAS-Cog scores at screening were 21 points (range 16–23) and 29 points (range 23–35), respectively. All patients were treated with ChEI as concomitant medications with a median duration of 17 months (range 5–60) before inclusion, and they remained on ChEI treatment throughout the study. All patients were treated for hypertension and were normotensive at the time of inclusion. Three patients had a medical history of depression, and one patient was taking levothyroxine for a hypothyroid condition but was euthyroid at inclusion. Brain glucose metabolism, assessed with PET imaging at baseline, was consistent with an AD diagnosis in all four patients. The AD diagnosis was confirmed histologically on Aβ- and tau-positive cortical biopsies from the implant site in all four patients (data not shown).

### Safety and tolerability

A total of 16 AEs were reported during the study. The most common AEs were mild post-operative headache and transient post-operative delirium (Table 1). No serious AE was reported, but one AE was of medical importance: a left sub-acute subdural haematoma (SDH) discovered incidentally by MRI at the 3-month follow-up. The haematoma was in a parieto-occipital location with local mass effect but no midline shift. The haematoma did not extend frontally to the two implants on the left side and did not displace the implants from their targeted positions in the basal forebrain. The patient developed slight fatigue, but no focal neurological signs were found. The haematoma was managed conservatively and resolved within 3 months. There was no indication of weight loss or back pain in any of the patients. No sign of blood-brain barrier dysfunction was found, based on CSF/albumin ratio assessment. All AEs were resolved by the end of the trial period at 6 months.

**Table 1:** Adverse events during the 6-month trial period

Adverse event	Number
Delirium	2
Headache	7
Hypertension	1
Subdural haematoma	1
Fatigue	1
Nasopharyngitis (common cold)	1
Abnormal clinical chemistry findings^a^	1
Nausea/vomiting	1
Herpes simplex blister	1
Total number	16

^a^Hypokalaemia

### Surgical procedures

All study participants underwent surgery according to the study protocol without complications. Post-operative cranial CT scans confirmed that NGF-ECB implants were positioned in the intended anatomical targets without adverse radiographic findings. Overall, the post-operative course was uneventful, and the surgical scalp incisions healed well in all patients. The implants remained in position during the study period and were easily removed by the end of the study without any complications or safety concerns. Implants were sent for NGF analyses and histology, and patients were discharged two days after surgery.

### Cognition and magnetic resonance imaging

Cognition was assessed on the basis of performance on clinical rating scales. At baseline, the median MMSE score was 21 (range 16–23), and the median ADAS-Cog score was 29 (range 23–35). All patients showed declines of 2–3 points on the MMSE (median 19, range 14–21) and increased by 3–6 points on the ADAS-Cog (median 33, range 26–41) during the study. Cognitive decline was paralleled by a decrease in mean BV/CSF index value [38] at 6 months (16.2 ± 0.7) compared with baseline (18.7 ± 1.6), without reaching statistical significance (*Z* = −1.826, *p* = 0.068). The patients were followed clinically for an additional 20 months after implant removal. Three patients continued to follow the same rate of decline in MMSE score after implant removal, while one patient showed a steeper rate of cognitive decline (data not shown).

### Cerebrospinal fluid analyses

**Fig. 3:**
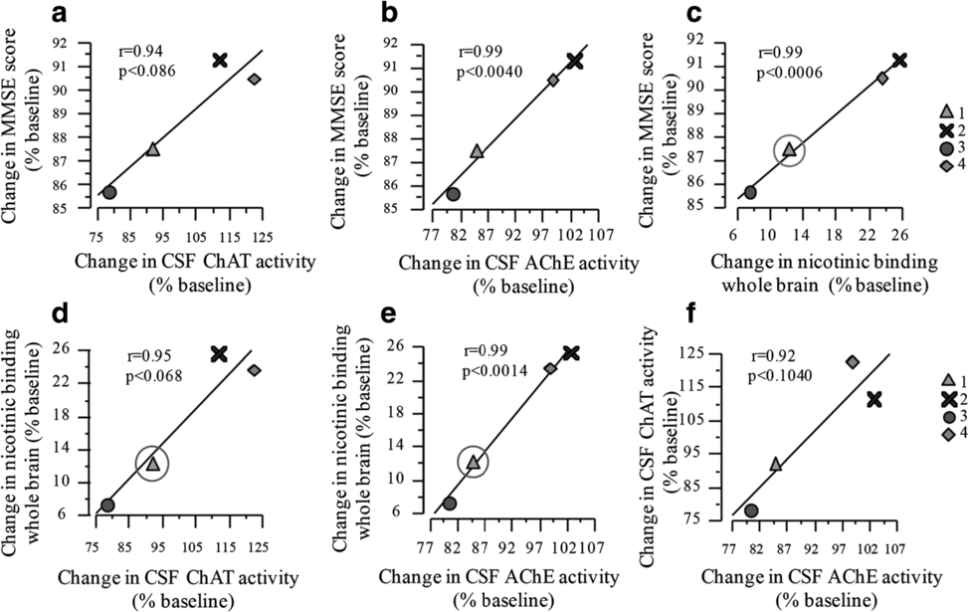
Percentage change from baseline in cerebrospinal fluid (CSF) activities of choline acetyltransferase (ChAT) and acetylcholinesterase (AChE) correlated positively with change from baseline in Mini Mental State Examination (MMSE) score (**a** and **b**) and nicotinic binding in whole brain (**d** and **e**), respectively. The baseline value for nicotinic binding for patient 1 was extrapolated from an average of baseline values from patients 2–4 and is indicated by a *circle*. Change from baseline in nicotinic binding correlated positively with change from baseline in MMSE score (**c**). Change from baseline in AChE activity correlated positively with change from baseline in ChAT activity (**f**)

No anti-NGF antibodies were detected in CSF at 3 or 6 months for any of the four patients, and NGF levels in CSF were below the detection limit of the assay (ELISA) at baseline and at 3 and 6 months. Compared with baseline, levels of Aβ, t-tau and p-tau did not change significantly at 3- or 6-month follow-up (Table 2). Levels of CSF NFL were increased in all patients at 3 months but had decreased at the 6-month time point, although not to baseline levels. This change in NFL levels was not paralleled by an increase in CSF GFAP (Table 2). CSF ChAT and AChE activities were increased in two of the four patients at 6 months compared with baseline. These increased activities coincided with a lesser decline in MMSE compared with the other two patients, and we found a significant positive correlation between change in MMSE score and change in ChAT or AChE activity from baseline to 6 months, respectively (Fig. 3a, b).

**Table 2:** Cerebrospinal fluid biomarkers at baseline and at 3 and 6 months

	BL, median (range)	3 months, median (range)	*p* Value vs. BL	6 months, median (range)	*p* Value vs. BL
Aβ_1–42_, ng/L	188 (134–208)	202 (113–207)	1.0	183 (123–206)	0.27
t-tau, ng/L	128 (70–235)	120 (64–256)	1.0	113 (76–187)	0.46
p-tau, ng/L	36 (18–76)	42 (35–48)	1.0	46 (33–55)	0.72
NFL, ng/L	301 (57–557)	1959 (846–2632)	0.07	718 (236–758)	0.07
GFAP, ng/L	586 (395–891)	612 (387–943)	0.72	589 (353–833)	1.0
ChAT activity, nmol/ml/minute	373 (317–447)	367 (279–414)	0.14	358 (337–410)	0.47
AChE activity, nmol/ml/minute	12 (9–22)	10 (9–21)	0.07	10 (9–23)	0.27

*Abbreviations: BL* baseline, *Aβ*_*1–42*_ amyloid-β peptide 1–42, *t-tau* total tau, *p-tau* phosphorylated tau, *NFL* neurofilament light chain protein, *GFAP* glial fibrillary acidic protein, *ChAT* choline acetyltransferase, *AChE* acetylcholine esterase

### In vivo ^11^C-nicotinic binding by positron emission tomography

We found no increase in regional ^11^C-nicotinic binding across individual patients. However, changes from baseline in ^11^C-nicotinic binding in whole brain correlated positively with changes in CSF ChAT and AChE activities (Fig. 3d, e). Furthermore, change from baseline in ^11^C-nicotinic binding in whole brain correlated significantly with change in MMSE score (Fig. 3c).

### Implant performance

**Fig. 4:**
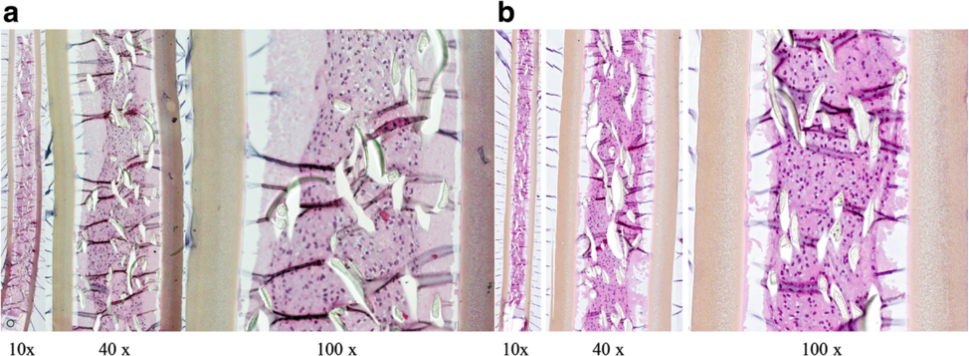
Histology from two nerve-growth-factor- encapsulated cell biodelivery implants taken from (**a**) patient 1 (implant number 4) and (**b**) patient 4 (implant number 23) after explant. Original magnifications × 10, ×40 and × 100, as indicated

**Fig. 5:**
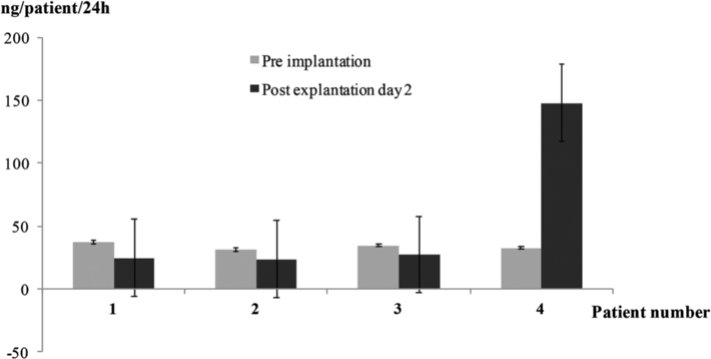
Mean release of nerve growth factor from nerve-growth-factor-encapsulated cell biodelivery implants represented in nanograms per patient per 24 h, before implant (pre-implant) and at day 2 after explant (post-explant day 2) in the four patients

Sixteen NsG0202.1 implants were selected for implanting. The selected implants released a mean ± SD of 8.6 ± 2.2 ng NGF/24 h pre-operatively, five times higher than the first-generation NsG0202 implant used for the first two dose cohorts [21]. All implants were retrieved at 6 months and proved to be intact with no adherence of inflammatory cells or connective tissue. Figure 4 shows the histology of implants number 4 (patient 1) and number 23 (patient 4) after explant. Viable cells can be seen in clusters between cell-supportive scaffolds inside the hollow membrane capsule. The majority of capsules were identified with continued secretion of NGF. All but 3 implants released NGF, and 9 of 16 devices released more NGF at the end of the study than at baseline. Following a peak value at day 1 following retrieval, the rate of NGF release stabilized at 24–48 h. On the basis of measures of sustained release in vitro at day 2, the values of total NGF released per patient per 24 h were 24.4 ng, 23.4 ng, 27.3 ng and 148.8 ng (Fig. 5).

## Discussion

We report on a dose escalation step of an ongoing phase I study of ECB-NGF to the cholinergic basal forebrain in patients with mild to moderate AD. The safety and tolerability of a second-generation NGF-ECB implant was explored in four patients for 6 months, and thereafter the implants were explanted. We demonstrate that stereotactic deep brain implant of NGF-ECB is feasible and safe and that sustained release of NGF to the target areas is well-tolerated. Implants demonstrated robust cell survival correlating with NGF release. Furthermore, we found a significant correlation between cognition and cholinergic markers in CSF and in brain, for change from baseline compared with the end of the study.

Due to suboptimal gene expression and limited cell survival in the first-generation implant at explant, we developed a second-generation NGF-ECB implant with several modifications for a dose escalation step. First, we replaced the polyvinyl alcohol scaffolding with a polyester terephthalate yarn that provided better support for the cells and improved cell survival (not shown) and manufacturability. To produce greater NGF secretion, we generated regulation-compliant, clonal, NGF-secreting ARPE-19 cell lines using an SB transposon expression system [39]. This technology uses a transposase that mediates genomic integration of multiple copies of the transgene inserted between two transposon terminal inverted repeats [27]. SB transposon-mediated gene delivery has been explored in several animal models of genetic disorders, as well as in a first-in-human application in oncology [40].

To our knowledge, our present study is the first reported clinical application of ex vivo SB transposon-mediated gene delivery to the CNS. The vector system is capable of stable gene transfer with long-term expression at an efficacy comparable with that of viral systems [41]. The NGC-0211 cell line showed at least a ten-fold higher NGF expression than the previously used cell line, NGC-0295, and secreted correctly processed bioactive NGF without any significant amount of pro-NGF. Together, these revisions yielded improved cell viability with a five-fold increase in NGF release compared with the first-generation implant. Following explant of implants at 6 months, the in vitro NGF analyses confirmed that all four patients had been administered NGF during the entire study, with three of the patients receiving similar doses, while three of four implants retrieved from one patient released higher NGF levels at explant than at implant, albeit without any adverse clinical findings.

Targeted delivery of NGF to the cholinergic basal forebrain was suggested as a potential disease-modifying treatment in 1990 [16], and the first clinical pilot study employed an ex vivo technique using NGF-secreting fibroblasts stereotactically implanted in the basal forebrain [10]. In one deceased study subject, histology of the basal forebrain confirmed trophic effects of NGF mirrored by sprouting of cholinergic neurons. A similar observation was made in a subsequent phase I study using the adeno-associated CERE-110 delivery vector, with post mortem pathology showing cholinergic hypertrophy 4 years after gene delivery [18]. In contrast to vector-based NGF gene delivery, the ECB technology has several advantages from a patient safety perspective. First, the grafted cells are encapsulated and isolated from the host cells, making it possible to remove the cells in case of complications and reducing the risk of graft-versus-host rejection. In addition, the implant can be replaced with a new or modified cell capsule as part of dose escalation or dose de-escalation using an indwelling catheter without stereotactic surgery [42]. Second, there is no genetic manipulation of host cells, minimizing the risk of mutations and carcinogenesis. Third, the risk of down-regulation of transgene expression is reduced because there is no physical contact between the host and encapsulated cells. Fourth, the secretion of NGF occurs along the entire length of the semi-permeable membrane, allowing for a controlled and potentially greater distribution of NGF to the basal forebrain than possible with viral vector gene delivery [43]. Cell encapsulation technology has advanced to become a viable therapeutic option for chronic disorders of the CNS [19].

Previous studies of NGF delivery to patients with AD have demonstrated improved cognition [10], cerebral glucose metabolism, nicotinic binding and electroencephalogram pattern [8, 12] in a subset of patients. In our present study, none of the patients showed improved cognition during the 6-month study period, which was expected, considering the short duration of the trial. However, there was no evidence of cognitive decline greater than expected for a 6-month period, also consistent with previous reports [44]. Levels of CSF AD biomarkers Aβ_β_ and tau proteins did not change significantly during the study period. Levels of CSF NFL, a marker of acute tissue damage [45], were increased in all patients at 3 months but had decreased at the 6-month time point, although not to baseline levels. This phenomenon was also observed in previous dose cohorts of this study [20]. In all cohorts in this study, a small cortical biopsy was taken for the purpose of diagnosis prior to implanting of the ECB device and would have contributed to the increase in CSF NFL levels. In a study of deep brain stimulation electrode implant in patients with Parkinson’s disease, researchers have also reported an associated increase in CSF NFL levels, a sign of limited acute brain tissue damage, immediately following electrode implant and then a decline over several months [46].

CSF levels of ChAT and AChE were similar to those detected in patients in previous dose cohorts of this study [23]. Consistent with our previous findings, we found a positive correlation between changes in MMSE during the study and changes from baseline in the three in vivo markers of cholinergic activity: (1) the acetylcholine synthesizer (ChAT), (2) degrading enzyme (AChE) activities and (3) nicotinic receptors. We found no significant positive effect on BV by MRI, while results derived from previous dose cohorts showed a slower rate of brain atrophy by MRI in a subset of patients during NGF administration [22]. However, a 6-month study period is probably too short to capture structural brain changes in patients with AD.

An SDH was the only clinically significant AE in this dose cohort and the second one reported in this study. Similarly to the previous SDH, it was diagnosed incidentally on the basis of the 3-month MRI and was managed conservatively. In both patients, the haematomas were localized away from the site of implant and did not dislocate the implants. Both patients continued to participate in the study and did not develop any sequelae related to the SDH.

## Conclusions

Safety and tolerability was demonstrated for the NGF-ECB technology with a second-generation device capable of sustained release of NGF in the majority of 16 implants at 6 months (3 implants did not release measurable amounts of NGF at explant). No AEs were deemed to be related to NGF or the device. These data support further development of ECB technology for treatment of AD and other neurodegenerative disorders. The design of implants is under further refinement to obtain a more predictable and stable long-term targeted NGF delivery to the brain in patients with AD. Future studies in AD need to be carried out for at least 18 months to better capture positive effects on the cholinergic system and associated cognitive improvement in randomised controlled trials.

## Abbreviations

AChE, acetylcholinesterase; AD, Alzheimer’s disease; ADAS-Cog, Alzheimer’s Disease Assessment Scale-Cognitive subscale; AE, adverse event; Aβ_1–42_, amyloid-beta peptide 1–42; BV, brain volume; ChAT, choline acetyltransferase; ChEI, cholinesterase inhibitor; CNS, central nervous system; CSF, cerebrospinal fluid; CT, computed tomography; ECB, encapsulated cell biodelivery; ELISA, enzyme-linked immunosorbent assay; GFAP, glial fibrillary acidic protein; GMP, Good Manufacturing Practice; MMSE, Mini Mental State Examination; MRI, magnetic resonance imaging; NFL, neurofilament light chain protein; NGF, nerve growth factor; PET, positron emission tomography; p-tau, phosphorylated tau 181; RPE, retinal pigment epithelial cell line; SB, Sleeping Beauty; SDH, subdural haematoma; t-tau, total tau
